# Nutritional Adequacy and Dietary Assessment Approaches in Institutionalised Older Adults Living in Long-Term Care Settings: A Systematic Review (2004–2024)

**DOI:** 10.3390/nu18010054

**Published:** 2025-12-23

**Authors:** Nicolás Piedrafita-Páez, Mª Angeles Romero-Rodríguez, Mª Lourdes Vázquez-Odériz, NUTRIAGE Study Researchers

**Affiliations:** Areas of Nutrition and Food Science and Food Technology, Department of Analytical Chemistry and Food Science, Faculty of Science, University of Santiago de Compostela, 27002 Lugo, Spain; nicolas.piedrafita@rai.usc.es (N.P.-P.); lourdes.vazquez@usc.es (M.L.V.-O.)

**Keywords:** long-term care, institutionalized older adults, dietary assessment, weighed food records, menu analysis, nutritional adequacy, micronutrient deficiencies

## Abstract

Background: Adequate nutrition in long-term care (LTC) settings is critical for the health and well-being of institutionalised older adults, yet global evidence consistently reveals significant gaps in dietary provision. Methods: We conducted a systematic review of observational studies published between January 2004 and December 2024 in PubMed and Scopus, following PRISMA 2020 and JBI guidelines. The review assessed whether planned menus and residents’ actual intake met recognised dietary reference values, described dietary assessment methods, and identified common nutrient shortfalls. Results: 34 observational studies from 16 countries were included. The most frequently used assessment methods were weighed food records (50.0%), menu analyses (29.4%), and 24 h recalls or food diaries (20.6%). Among the 25 studies reporting mean daily energy intake, 68.0% documented values between 1250 and 1800 kcal/day, and 73.5% indicated intakes below established reference values. Additionally, 11 studies (32.4%) found that residents consumed less than 75% of the energy planned in menus. Protein intake was below 60 g/day or 0.83 g/kg body weight/day in 41.2% of studies. Across 22 studies assessing micronutrients, recurrent inadequacies included vitamin D (61.8%), calcium (55.9%), folate (50.0%), zinc (41.2%), and fibre (47.1%). In studies quantifying planned–served–consumed stages, actual intake represented approximately 64.0–87.0% of planned energy and protein. Conclusions: Nutrition in LTC settings frequently falls short of meeting the energy and nutrient requirements of institutionalised older adults. Persistent inadequacies in energy, protein, and key micronutrients were observed across studies, alongside substantial variability in dietary assessment methods and reference frameworks, limiting comparability of findings.

## 1. Introduction

Ensuring adequate nutrition in long-term care (LTC) settings remains a critical challenge for institutionalised older adults [1,2]. Food provision is essential to maintain residents’ health and functional status, yet international evidence consistently reports insufficient intake of energy, protein, and micronutrients in this population [3,4,5,6,7]. Despite the psychosocial and environmental dimensions of mealtimes in LTC, the present review focuses specifically on nutritional aspects: the adequacy of food provision and intake, the methods used to assess them, and the discrepancies between planned menus and actual consumption. The issue of ensuring adequate nutritional provision and intake in LTC remains a significant challenge. Numerous studies document deficits in energy, protein, and key micronutrients—often exacerbated by limited mealtime support among residents with physical limitations, cognitive impairment, or texture-modified diets [4]. These factors contribute to discrepancies between the nutritional content of planned menus, the food served, and what residents ultimately consume.

The assessment of nutritional requirements in LTC is a methodologically complex undertaking. Typically, menus are planned at the facility or group level, whereas intake is determined at the individual level. This is influenced by appetite, preferences, health status, and assistance during meals. Previous studies have generally examined either menu composition or individual intake. These studies have utilised a variety of methods in their analysis, including the following: weighed food records, menu analysis, 24 h recalls, food diaries, food-frequency questionnaires. Furthermore, these studies have employed diverse reference frameworks, encompassing Dietary Reference Intakes, EFSA Dietary Reference Values, Nordic Nutrition Recommendations, and national guidelines [8,9,10,11]. This methodological heterogeneity limits comparability and hampers a unified interpretation of nutritional adequacy in LTC settings. In this review, “nutritional adequacy” is defined as the extent to which planned menus and/or actual intake meet recognised dietary reference values for energy, macronutrients, and micronutrients in older adults.

It is evident that there are significant lacunae in the extant literature. No previous synthesis has integrated findings across the planned–served–consumed continuum, systematically compared dietary assessment methods, or examined how different reference frameworks influence adequacy conclusions. A comprehensive review addressing these elements is therefore required.

The present systematic review is concerned with the following question: to what extent do planned menus and actual dietary intake in LTC settings meet recognised dietary reference values for institutionalised older adults, and which dietary assessment approaches and reference standards are used to evaluate this adequacy? The objectives that were set out were as follows: firstly, summarise the nutritional adequacy of planned menus and residents’ actual intake; secondly, characterise the dietary assessment approaches used; and thirdly, identify the nutrients most frequently reported as inadequate and the dietary reference frameworks applied, including studies reporting planned, served and consumed values.

## 2. Materials and Methods

### 2.1. Design and Methodological Approach

**Figure 1:**
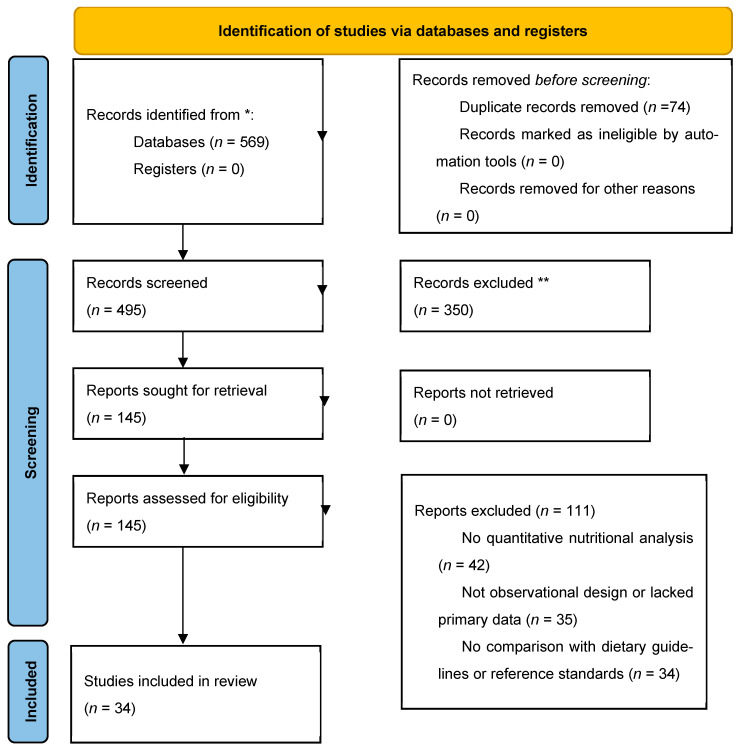
PRISMA Flowchart of Study Selection. * Records identified from PubMed and Scopus; ** Records excluded during the initial screening of titles and abstracts.

A systematic review was conducted in accordance with Joanna Briggs Institute methodology for observational cross-sectional studies and following the PRISMA 2020 statement [8,9]. The objective of the review was to synthesise evidence on the nutritional adequacy of menus and actual intake in LTC settings, the dietary assessment methods applied, and the extent to which provision meets recognised dietary reference values. For the purposes of this review, the term “nutritional adequacy” was defined as the extent to which planned menus and/or consumed intake meet established reference values for energy, macronutrients and micronutrients. This systematic review was registered in PROSPERO (record ID: 1265793). The selection of studies is illustrated in the PRISMA flow diagram (Figure 1), and the PRISMA checklist is provided in Supplementary Table S1. Full electronic search strategies are detailed in Supplementary Table S2. The timeframe selected for this study (2004–2024) was determined by the widespread adoption of major international dietary reference frameworks that occurred in the early 2000s. The most recent database search was updated in early 2025, yet no additional eligible studies published after December 2024 were identified. The term “LTC settings” was defined as follows: nursing homes, residential aged care homes, skilled nursing facilities, service housing and other comparable 24 h care environments with centrally planned meal provision. All the facilities included were subject to the same institutional food-service model. This review constitutes a component of the NUTRIAGE research project, which develops nutrition-based strategies for healthy ageing in the Galicia–North Portugal Euroregion (ref.: 0659_NUTRIAGE_1_E).

### 2.2. Study Search and Selection Strategy

A comprehensive search was conducted in PubMed and Scopus for studies published between January 2004 and December 2024. The commencement year was selected because significant international dietary reference frameworks (e.g., updated DRIs, Nordic Nutrition Recommendations) had become extensively adopted in the early 2000s; the concluding date denotes the most recent update prior to the manuscript revision. The selection of PubMed and Scopus was made on the basis of their extensive coverage of nutrition and gerontology research; Web of Science, Embase and CINAHL were excluded due to limitations in resource and preliminary scoping indicating minimal additional yield.

The search strategy encompassed a combination of controlled vocabulary and free-text terms, with a focus on institutionalised older adults and LTC settings (e.g., “older adults”, “nursing homes”, “long-term care”, “care homes”, “residential care”), food provision and dietary assessment (e.g., “menu planning”, “dietary assessment”, “nutritional adequacy”, “nutrient intake”, “diet quality”, “energy intake”, “protein intake”, “micronutrient adequacy”, “plate waste”), and reference standards (e.g., “dietary guidelines”, “reference values”, “nutritional standards”). An example PubMed string was: (“Aged” [Mesh] OR elderly [Title/Abstract] OR “older adult *” [Title/Abstract]) AND (“Nursing Homes” [Mesh] OR “Long-Term Care” [Mesh]) AND (“menu planning” [Title/Abstract] OR “nutritional adequacy” [Title/Abstract] OR “dietary assessment” [Title/Abstract]). Only peer-reviewed articles in English or Spanish were included; grey literature (theses, reports and book chapters) and conference abstracts were excluded. Studies that were confined to a single mealtime (e.g., lunch or dinner) were considered eligible if nutrient estimates permitted comparison with daily reference values; however, none of the included studies fulfilled this condition. The screening process was conducted in three stages (titles, abstracts, full texts) by a single reviewer, with subsequent verification by a second researcher. No discrepancies were identified during this process. Furthermore, reference lists of included studies and relevant reviews were meticulously hand-searched to identify additional records. In instances where full texts were not accessible via institutional subscriptions, attempts were made through inter-library loan. It is important to note that no automation tools or machine-learning classifiers were utilised in the process.

### 2.3. Inclusion and Exclusion Criteria

The inclusion and exclusion criteria for this review are summarised in Table 1.

The eligibility criteria were defined a priori and applied consistently during all screening phases (titles, abstracts, full texts) in accordance with PRISMA guidance. The present study incorporated observational studies (cross-sectional or longitudinal) that quantitatively analysed food provision or dietary intake in institutionalised older adults (≥60 years) residing in LTC settings. The energy and/or nutrient intake of these individuals was then compared with recognised dietary reference values or guidelines (e.g., DRIs, EFSA DRVs, NNR, ESPEN or national recommendations).

For the purposes of this review, the term “nutritional analysis” was defined as any quantitative estimation of the energy and/or nutrient content of planned menus, food served or actual intake. Studies reporting exclusively descriptive menu information devoid of nutrient calculations, were excluded from the analysis. Qualitative or mixed-methods studies were eligible for inclusion only if they included quantitative dietary analysis; however, none were identified.

Studies assessing only part of the menu (e.g., lunch or dinner) were included if nutrient estimates allowed comparison with daily reference values. In the course of the present study, a decision was taken to exclude the following categories of studies: those conducted in hospitals, acute-care clinics, day-care centres or among community-dwelling populations; those focused solely on satisfaction or perceptions without quantitative nutritional analysis; reviews without primary data; and non–peer-reviewed sources. Grey literature, including theses, reports, and book chapters, as well as conference abstracts, were not considered in this study.

### 2.4. Extraction and Synthesis of Data

The data from each included study were extracted by one reviewer using a predesigned template and checked for accuracy and completeness by a second researcher. Any uncertainties were resolved through discussion. A comparative evidence table (Table 2) was compiled, integrating the main characteristics and results of the included studies These characteristics and results are as follows: setting and country, year of publication, study design, assessment method used, principal findings (energy, protein and micronutrient intakes), comparison with dietary guidelines and reference values, and the main strengths and limitations reported by the authors. The dietary assessment methods encompassed a range of techniques, including direct duplicate weighing (weighed food records), standardised visual estimation of plate waste, 24 h dietary recalls, food diaries and food frequency questionnaires (FFQs). These were complemented by menu analyses using validated nutrition software packages (e.g., NutriSurvey^®^, DietPro, among others) and national food composition tables.

When information was incomplete or inconsistent, a conservative approach was applied: non-reported variables were coded as “NR” in the evidence table, and no statistical imputation was undertaken. Given the substantial heterogeneity in dietary assessment methods (e.g., weighed records, visual plate-waste estimation, recalls, diaries, FFQs, menu analyses), settings and reference frameworks, no meta-analysis was attempted; instead, results were synthesised narratively, focusing on the direction and magnitude of energy, protein and micronutrient inadequacies and on discrepancies between planned, served and consumed levels.

### 2.5. Methodological Quality Assessment

The methodological robustness of the included studies was evaluated using the Joanna Briggs Institute (JBI) Critical Appraisal Checklist for Analytical Cross-Sectional Studies, which was specifically designed for observational cross-sectional research [8]. The checklist comprises eight items that assess key aspects of internal validity, including clarity of inclusion criteria, validity and reliability of measurements, identification and control of potential confounders, and the appropriateness of statistical analyses. Each item was rated as “yes”, “no”, “unclear” or “not applicable”, and total scores were calculated by summing the number of “yes” responses. As JBI does not specify cut-offs, we adopted an operational classification agreed by the review team: ≥7/8 items = high quality, 5–6/8 = moderate, ≤4/8 = low quality. Individual scores are shown in Table 2. The comparative synthesis table (Table 2) presents the individual ratings for each study. The appraisal was conducted by the first author and verified by senior investigators; no disagreements occurred. No additional formal risk-of-bias tool was applied. The potential subjectivity of this process is acknowledged in the Discussion.

## 3. Results

### 3.1. Study Characteristics

A total of 34 observational studies published between 2004 and 2024, all conducted in institutional LTC settings, predominantly nursing homes, but also including comparable environments such as service housing with 24 h care and centrally planned meal provision, as defined in Section 2.1. During full-text screening, 111 reports that initially appeared to meet the inclusion criteria were excluded for the following reasons: lack of quantitative nutritional analysis (*n* = 42), non-observational design or absence of primary data (*n* = 35), and lack of comparison with dietary guidelines or nutritional reference standards (*n* = 34). The PRISMA 2020 flow diagram (Figure 1) details the overall selection process and exclusion steps. Eligibility criteria applied during study selection are summarised in Table 1, while key study characteristics (country, setting, design, sample size, age and primary focus), nutritional adequacy findings, dietary assessment approaches and methodological quality (JBI scores) are presented in Table 2.

Sample sizes ranged from 25 to 639 residents in studies with individual data and covering 16 countries across Europe, the Americas, Asia and Oceania. Canada was the leading contributor, with nine studies, followed by Spain (*n* = 5), Australia (*n* = 4), Belgium (*n* = 2), Finland (*n* = 2), Indonesia (*n* = 2) and Poland (*n* = 2). One study was conducted in each of France, Denmark, Croatia, Türkiye, Sweden, Slovenia, New Zealand and Brazil. The distribution of publications over time was as follows: seven studies were published between 2004 and 2010, ten between 2011 and 2015, nine between 2016 and 2020, and eight between 2021 and 2024. Most investigations adopted cross-sectional observational designs; a notable exception was the longitudinal comparative study by Jyväkorpi et al., which analysed two cohorts a decade apart (2007 and 2017–2018) [10].

In the cases where data was reported, the population under study was predominantly composed of individuals aged ≥ 80 years, with a marked predominance of women and a high prevalence of multimorbidity, functional dependency and risk of malnutrition or frailty. The assessment of these factors frequently involved the utilisation of tools such as the Mini Nutritional Assessment. Several studies also incorporated specific subgroups, including residents with dementia, those requiring texture-modified diets, and individuals at high risk of sarcopenia. However, the clinical and functional characteristics (e.g., degree of cognitive impairment, level of assistance at meals, polypharmacy) were not consistently described across all studies, which precluded the derivation of aggregated summary statistics for these variables at review level.

The investigations converged on five principal lines of enquiry. Initially, a series studies evaluated were conducted to evaluate the actual intakes of energy, macronutrients and micronutrients at the resident level. Secondly, a series of studies analysed the nutritional content of planned menus and compared it with official reference frameworks. Thirdly, a few studies evaluated the overall quality of the menu in terms of food-group composition, dietary patterns and nutrient density. Fourthly, others examined the links between diet and nutritional status, using indicators such as malnutrition, sarcopenia, frailty, body mass index or calf circumference. Finally, a subset of investigations explored contextual determinants of nutritional adequacy, including plate waste, texture-modified diets, mealtime environment and polypharmacy, and in some cases the cost or efficiency of protein-enrichment strategies. Despite the fact that the reference frameworks employed (e.g., DRIs from the Institute of Medicine, EFSA Dietary Reference Values, national guidelines, WHO recommendations, ESPEN/PROT-AGE criteria) are characterised by preventive orientation, they differ in structure and nomenclature (e.g., RDA, AI, EAR, PRI). Throughout this manuscript, the term “nutritional references” is used to encompass these official frameworks from recognised authorities.

### 3.2. Methodological Quality

The JBI appraisal revealed that 10 out of 34 studies (29.4%) were classified as high quality (7–8/8 items), while the remaining 24 (70.6%) were designated as moderate quality (4–6/8); It is noteworthy that none of the studies were deemed to be of low quality (≤3/8). Overall, 32 studies (94.1%) scored ≥6/8, indicating a predominantly moderate-to-high methodological quality (Table 2).

The most frequently unmet checklist items were: (i) identification and control of potential confounders (18/34; 52.9%), (ii) explicit sample-size justification (20/34; 58.8%), and (iii) complete description or validation of dietary assessment tools (7/34; 20.6%). Variables such as functional dependency, cognitive impairment, need for mealtime assistance, texture-modified diets and polypharmacy were often reported descriptively but not consistently incorporated as confounders.

In contrast, the majority of studies adhered to JBI criteria concerning outcome measurement and exposure assessment. The evaluation of dietary intake and menu content was conducted using recognised methods (e.g., weighed food records, structured visual plate-waste estimation, 24 h recalls or food diaries). In addition, several investigations utilised these with anthropometric measures, nutritional screening tools and/or biochemical markers to enhance the comprehensiveness of the research.

### 3.3. Dietary Assessment Methods

The predominant dietary assessment approach was weighed food records, used in 17 out of 34 studies (50.0%), with observation periods ranging from 1 to 21 days and particularly common in European countries such as Belgium, Spain, Finland, Poland and France [7,12,14,15,16,20,21,25,26,27,29,30,31,32,35,36,42]. A second group of 5/34 studies (14.7%) utilised 24 h dietary recalls administered via structured interviews, supported by photographs, household measures or digital tools, more frequently in Türkiye, Croatia, Poland and Indonesia [17,19,24,34,39]. Two studies (2/34; 5.9%) utilised food-frequency questionnaires (FFQ) or self-administered food records, either in institutionalised samples or within longitudinal cohorts [19,39]. Finally, 10/34 studies (29.4%) focused exclusively on theoretical analysis of the nutritional content of institutional menus, evaluating standardised recipes, technical sheets and portion sizes defined by kitchen or catering services [1,10,18,22,23,28,34,38,40,41].

A range of complementary techniques were also employed. Standardised visual assessments of plate waste were employed in 5/34 studies (14.7%) to estimate the proportion of food not consumed and to derive actual intake from what was served [12,21,31,38,41]. One study (1/34; 2.9%) applied a statistical modelling approach, the Multiple Source Method (MSM), to address intra-individual variability in repeated 24 h recalls [30]. Eight studies (8/34; 23.5%) incorporated clinical and/or biochemical indicators as part of the assessment of nutritional status. These indicators included anthropometry (body mass index, calf circumference), nutritional screening tools and serum micronutrient levels—as part of the assessment of nutritional status [7,10,20,24,31,32,33,42]. A structured food-service audit was employed in 1/34 studies (2.9%), with checklists evaluating aspects such as presentation, temperature, texture, variety and dining-room conditions [26]. Furthermore, 2/34 studies (5.9%) examined economic and sustainability dimensions, including the cost of protein-enrichment strategies, the impact of portion size on plate waste and the efficiency of food procurement [33,41].

### 3.4. Comparison with Dietary Guidelines/References

In a total of 12/34 studies (35.3%), IOM DRIs were utilised as the primary comparator, with subcategories (EAR, RDA, AI, UL) being applied in accordance with the nutrient under evaluation and the available evidence [11]. 18/34 studies (52.9%) relied on national food-based or nutrient-based guidance. This included Canada’s Food Guide [22], Spanish recommendations [43] and guidance from Türkiye, Indonesia, Slovenia, France, Poland and Belgium. A number of further references that were considered, including the European Food Safety Authority Dietary Reference Values (DRVs) [44], the Nordic Nutrition Recommendations (NNR) [45] and the Australia/New Zealand Nutrient Reference Values (NRVs) [46]. Three investigations incorporated World Health Organization (WHO) guidance [47], while two used clinical criteria from the European Society of Parenteral and Enteral Nutrition (ESPEN) [48] and the PROT-AGE group [49]. Furthermore, a few studies have employed qualitative frameworks, including the German Nutrition Circle [50], combining quantitative and qualitative approaches to institutional menu assessment. In accordance with the stipulated inclusion criteria, all studies made comparisons between their findings and at least one official nutrient-based or food-based framework; in several cases more than one framework was applied in parallel (see Table 2).

### 3.5. Nutritional Adequacy

As demonstrated in Table 2, the findings on nutritional adequacy exhibited variation according to the level of dietary assessment (i.e., planned menu analyses, food served/provision estimates, and actual intake); consequently, the results are interpreted in accordance with the original assessment level reported in each study. Of the 34 studies, 25 (73.5%) explicitly reported a mean daily energy estimate, either as actual intake or derived from served/provision-based assessments (see Table 2). Of the 25 studies, 17 (68.0%) reported mean intakes in the range of approximately 1250–1800 kcal/day, while 8 (32.0%) reported mean intakes >1800 kcal/day [1,18,22,23,28,34,39,42]. 11 (32.4%) studies indicated that actual consumption did not reach 75.0% of the available provision (planned or served), where provision-to-consumption comparisons were reported (Table 2) [12,13,14,15,16,21,25,27,31,35,41]. In studies directly comparing the consumption of food with its provision, the actual energy intake represented between 64.0% and 87.0% of the planned content [29,31,35]. In a particular study, it was found that only 20.0% of the dishes analysed satisfied predefined nutrient-density thresholds [41]. Collectively, these findings indicate that, across studies reporting energy estimates, mean values were most often ≤1800 kcal/day, and that provision-to-consumption gaps were frequently observed in studies directly comparing planned/served provision with consumption.

In 14 out of 34 studies (41.2%) mean protein intakes below 0.83 g/kg/day or 60.0 g/day, the EFSA population reference intake for adults [44,51]. For instance, Nanayakkara et al. documented that residents consumed only 69.0% of the planned protein [31]. In Buckinx et al., although 93.9% of the protein provided was consumed, the final mean intake (54.8 g/day) remained insufficient to cover the group’s needs [29]. In the course of investigations that examined protein provision per main meal, it was found that only a minority of meals reached the ≥25–30 g threshold that is often proposed for older adults.

With regard to the distribution of macronutrients (with the exception of protein), 17 out of 34 studies provided specific data on the relative energy contributions of fat and carbohydrate, whereas 17 out of 34 did not report these explicitly [10,13,17,18,19,20,21,22,25,27,28,29,32,33,36,41]. Among the subjects who provided such reports, fat contributed between 28.0% and 55.0% of the energy, while carbohydrate contributed between 34.0% and 61.0% [1,12,16,23,36]. Certain studies furnished absolute values: Woods et al. reported mean fat intakes of 57.0 g/day (women) and 68.0 g/day (men), and carbohydrate intakes of 248.0–261.0 g/day [15]. Lavrisa et al. documented mean saturated-fat intakes of 34.3 ± 10.1 g/day in men and 30.4 ± 8.3 g/day in women, associated with low intakes of wholegrains and dietary fibre [39]. It is evident that other studies have reflected distributions that are more closely aligned with recommendations, albeit with limitations in food quality. For instance, in the case of NH in Galicia and Portugal, Piedrafita et al. observed that carbohydrates accounted for 56.0% and 60.5% of energy, respectively, while fat contributed 24.0% and 20.4% [42].

With regard to micronutrients, 22 out of 34 studies (64.7%) provided specific data derived from either actual intake assessments or the content of institutional menus/records; 12 out of 34 studies (35.3%) did not report micronutrient data in sufficient detail. Within the full sample of 34 studies, reported deficiencies in calcium appeared in 11 (32.4%) studies, vitamin D in 10 (29.4%), dietary fibre in 9 (26.5%), folate in 8 (23.5%) and magnesium in 6 (17.6%). Zinc and potassium deficits were each described in 3 (8.8%) studies. In addition, isolated reports documented low intakes of vitamin A, vitamin C, vitamin E, vitamin B12, iron and iodine (1/34; 2.9% for each of these micronutrients). With respect to excesses, sodium intakes above recommended levels were noted in 4/34 (11.8%) studies and phosphorus in 1/34 (2.9%), particularly in menu analyses with a high use of processed meats, cheeses and bread products. As illustrated in Table 2, the micronutrients that deviated from reference values (by deficit or excess) are indicated, along with the comparative framework that was utilised.

## 4. Discussion

### 4.1. Overview of Nutritional Adequacy in Long-Term Care

This review synthesised the findings of 34 observational studies conducted across 16 countries, revealing a consistent pattern of nutritional inadequacy in institutional LTC settings. Most studies documented energy intakes that fell below recommended levels, with values infrequently exceeding the established thresholds. Furthermore, a substantial proportion of the studies indicated protein intake that was below the reference standards. It has been frequently observed that there are recurrent deficits in essential micronutrients, including but not limited to vitamin D, calcium, folate, zinc, magnesium, and dietary fibre. These are often accompanied by excessive sodium intake. However, only a minority of studies suggested simultaneous adequacy of energy, protein, and key micronutrients [12,16,21,27,29,31,32,35,36,38,40,41,42].

An additional finding was the gap between planned, served, and consumed intake. Several studies showed that residents consumed only a fraction of the theoretical provision, particularly in terms of energy and protein. This phenomenon was particularly pronounced among individuals with cognitive impairment, high dependency, or those following a texture-modified diet [7,29,31,41,42]. This phenomenon, termed “nutrient decay” underscores the notion that menu-level adequacy does not guarantee effective nutritional delivery.

In terms of methodology, the majority of, investigations employed cross-sectional designs and primarily relied on weighed food records, double weighing, or documentary menu analysis, supplemented in some cases by visual plate-waste estimation, 24 h recalls, food diaries, or FFQs [1,10,12,13,15,16,17,18,19,21,22,23,25,26,27,28,30,31,32,34,35,36,37,38,39,40,41,51]. The JBI appraisal indicated moderate-to-high methodological quality in 32 out of 34 studies (scores ≥ 6/8), suggesting that the overall signal of inadequacy is unlikely to be explained solely by study limitations. However, the utilisation of heterogeneous nutritional reference frameworks (DRIs, EFSA DRVs, WHO, national guidelines, ESPEN/PROT-AGE) and variable cut-offs [11,23,26,29,36,43,44,45,46,47,48,49,52] complicates direct comparison of numerical adequacy estimates across settings.

These findings highlight the need for strategies that integrate real intake assessment and menu optimisation to ensure nutritional adequacy in LTC environments.

### 4.2. Energy, Protein and Micronutrient Adequacy in LTC Residents: Comparison with Previous Evidence

When considered in the context of previous research on nutrition in LTC, these findings serve to reinforce longstanding concerns regarding undernutrition in institutionalised older adults. Earlier studies and reviews have primarily focused on the prevalence of malnutrition, anthropometric decline, and the use of oral nutritional supplements in single facilities or countries [7,10,23,26,42,48,52], and they similarly describe low energy and protein intakes together with frequent deficits in vitamin D, calcium, folate and zinc in LTC populations [13,16,23,27,29,36,37,51].

The findings of this review are also consistent with clinical guidelines that emphasise the challenge of attaining recommended protein intakes (1.0–1.2 g/kg/day, or 1.2–1.5 g/kg/day in cases of frailty or illness) in older adults who exclusively rely on standard menus [48,49,51]. The observation that only a minority of main dishes provide ≥25.0 g of protein [41] mirrors ESPEN and PROT-AGE recommendations, which emphasise the need for sufficient protein per meal to stimulate muscle protein synthesis [48,49]. Similarly, the recurrent deficiencies in vitamin D, calcium and other micronutrients are consistent with prior reports of pervasive insufficiency in institutional settings, even among resident’s r who appear to be receiving sufficient nutrition [13,23,27,29,36,37,51].

A distinctive contribution of this review is the explicit quantification of the planned–served–consumed gap across multiple countries and study designs. As previously outlined in the literature, the phenomenon of “nutrient decay” has been documented in individual cohorts [31,35,42]. However, there is a paucity of syntheses that have systematically contrasted menu-level provision with intake-level data. Furthermore, there is a dearth of studies that have related these patterns to methodological choices and reference frameworks. The present review assists in elucidating the reasons why estimates of adequacy, based exclusively on planned menus, frequently appear to be more favourable than those based on weighed intake, particularly in residents with high dependency, cognitive impairment or texture-modified diets [7,12,16,21,29,31,32,35,36,38,40,41,42].

Finally, the predominance of menu analysis and single-method approaches observed here is in line with earlier reports highlighting the logistical challenges of implementing gold-standard methods such as double weighing in routine practice [30,32,39,53]. Concurrently, the restricted utilisation of mixed method designs, coupled with the heterogeneity of normative frameworks corroborates earlier observations that the LTC nutrition literature is heterogeneous and methodologically diverse, thereby impeding synthesis and the extrapolation of results to more extensive populations [23,26,29,48,52].

### 4.3. Implications for Menu Design, Micronutrient Density and Nutritional Monitoring in LTC

When considered as a whole, the findings have several practical implications for the design and monitoring of food provision in LTC settings. Firstly, the combination of low mean energy intake and frequent failure to reach 75.0% of the energy theoretically provided by menus suggests that reliance on planned menus alone is insufficient to guarantee adequate intake, especially in residents with multimorbidity and high dependency. The accuracy of nutritional monitoring in LTC settings can be enhanced by conducting periodic assessments of actual consumption. This can be achieved with weighed food records, simplified double weighing or standardised visual plate-waste estimation in high-risk groups [12,16,21,27,29,31,32,35,36,38,40,41,42].

Secondly, the observed pattern of protein intake observed across studies indicates that both total provision and intraday distribution require explicit consideration. The finding that only 20.0% of main dishes provided ≥25.0 g of protein [41] suggests that current menus offer limited opportunities to reach the per-meal thresholds recommended to stimulate muscle protein synthesis [48,49]. In practical terms, these findings suggest that incorporating protein-enriched dishes and snacks could help meet recommended intakes. Greater inclusion of protein-rich dairy products, eggs, legumes and lean meats, and attention to the distribution of protein across breakfast, lunch, dinner and between-meal offerings, particularly for residents with sarcopenia, dementia or texture-modified diets, is also recommended [7,25,28,30,39,42].

Thirdly, the evidence of recurrent micronutrient deficits, in addition to the fact that certain menus remain below daily requirements even when planned quantities are consumed, as referenced in [13,23,27,36,37,51], indicates the importance of ingredient choice and menu composition. The incorporation of pulses, wholegrains, fresh fruit and vegetables, and fortified dairy products, into catering systems represents a pragmatic approach to ensuring the preservation of the micronutrient profile of standard menus [23,27,28,36,39]. In view of the recurrent excess sodium intake, a systematic review of salt use and processed foods is also indicated [13,23,29,34,36].

Fourthly, the heterogeneity of dietary assessment methods observed in this review suggests that LTC providers should adopt pragmatic, context-adapted combinations of tools rather than relying on a single approach. Double weighing has been identified as a suitable method for audits and research purposes. Conversely, documentary menu analysis, visual plate-waste estimation and periodic 24 h recalls, or food diaries may be more feasible for ongoing monitoring [19,25,30,31,32,35,39]. Frameworks such as the Quality Index for Nutrition in Nursing Homes (QUINN) illustrate how menu and food-service audits can be integrated into structured evaluation systems [54], although such tools are not yet widely implemented.

### 4.4. Methodological Considerations and Limitations of the Evidence

The synthesis presented here is subject to limitations at both the primary evidence and review levels. At the evidence level, the majority of included studies were of a cross-sectional nature, which precludes the possibility of causal inference regarding the impact of menu quality or intake patterns on clinical outcomes. However, mixed-method designs integrating planned menus, actual intake, and outcomes such as nutritional status, functional performance, or mortality were comparatively infrequent [29,30,39]. There was considerable heterogeneity in the dietary assessment methods employed (e.g., weighed food records, menu analysis, 24 h recalls, food diaries, FFQs, plate-waste estimation) and in the outcomes reported, with many studies focusing on a subset of nutrients or meals [1,10,12,13,15,16,17,18,19,21,22,23,25,26,27,28,30,31,32,34,35,36,37,38,39,40,41,51]. In contrast to the more systematic exploration of energy and protein adequacy, micronutrient adequacy has received less attention, despite the prevalence of deficiencies in vitamin D, vitamin B12, folate, calcium, and zinc [13,16,23,27,29,36,37,51]. The organisational determinants of nutritional outcomes, such as dietitian involvement, staff training, and food-service governance, have received scant attention in a structured manner, impeding the capacity to establish a correlation between institutional characteristics and nutritional outcomes [23,26,48,52]. Longitudinal data on the functional impact of food-based interventions in LTC remain scarce [10,38,53].

Despite the fact that 94.1% of studies demonstrated moderate-to-high methodological quality (achieving a minimum of ≥6/8 items on the JBI checklist), common limitations, including the absence of control for confounders and the lack of a sample-size justification, have the potential to influence absolute adequacy estimates. To illustrate this point, failure to adjust for functional dependency or cognitive impairment has the potential to underestimate intake in highly dependent residents. Conversely, the omission of mealtime assistance variables may lead to overestimation. However, the uniformity of energy and protein deficits observed across various methodologies, geographical locations, and analytical frameworks indicates that these patterns are unlikely to be artefacts of study design. The observed heterogeneity primarily affects the comparability of numerical estimates rather than the direction of effect, which remained strongly indicative of recurrent inadequacies.

It is imperative that a few constraints are recognised at the review level. The search was restricted to the PubMed and Scopus databases, and to peer-reviewed articles in English or Spanish. It is possible that relevant studies indexed elsewhere or published in other languages were not identified. The protocol was not prospectively registered, and substantial heterogeneity in outcomes, dietary assessment methods, and reference frameworks precluded meta-analysis and necessitated narrative synthesis. The screening and data extraction processes were conducted by one reviewer and verified by a second, which may have introduced selection or extraction bias despite efforts to ensure consistency. Finally, since each study adhered to the specified nutritional reference framework and cut-offs, the numerical estimates of adequacy are not directly comparable across studies. Consequently, these should be interpreted as patterns and directions of effect rather than interchangeable percentages.

### 4.5. Future Research Priorities in LTC Nutrition

The gaps identified in this review suggest several research priorities for future investigation. Firstly, longitudinal and intervention studies are required to quantify the impact of specific food-based strategies on nutritional adequacy and clinical outcomes in LTC residents. Such strategies may include energy–protein fortification, optimisation of texture-modified diets, redistribution of protein across meals, and structured mealtime assistance [10,29,30,38,39,53]. Secondly, there is a need for pragmatic assessment protocols that combine menu-level and intake-level data with clinical indicators of nutritional status, using feasible methods for residents with cognitive impairment and high dependency. Thirdly, the development of LTC-specific nutritional reference frameworks tailored to institutionalised older adults would improve the comparability and clinical relevance of adequacy estimates [23,26,48,52]. Finally, it is imperative to employ robust designs when evaluating organisational determinants such as dietitian involvement, staffing models and food-service governance. These determinants have the potential to influence not only the quality of food provision but also the capacity to implement effective nutritional interventions [7,23,42,48,52]. Addressing these areas would serve to strengthen the evidence base for person-centred, nutritionally adequate food provision in LTC settings.

## 5. Conclusions

This systematic review indicates that, in the included LTC studies, energy and protein intakes frequently fall below recommended levels. Furthermore, deficits in key micronutrients such as vitamin D, calcium, folate, zinc and fibre are common, even when planned menus appear adequate. At the same time, there is considerable heterogeneity in both the dietary assessment methods employed to estimate intake and the nutritional reference frameworks utilised to evaluate adequacy. This limits the comparability across studies and hinders the translation of evidence into routine practice. A consistent discrepancy between planned, served and consumed food indicates that menu-level adequacy does not necessarily translate into effective nutritional delivery, particularly in clinically vulnerable subgroups and among those receiving texture-modified diets. These findings emphasise the necessity for geriatric-specific nutritional reference frameworks and pragmatic, mixed-method monitoring strategies that integrate menu provision and actual intake to support more reliable and person-centred nutritional care in LTC settings.

## Figures and Tables

**Table 1:** Inclusion and exclusion criteria.

Category	Inclusion	Exclusion
Population	Institutionalised older adults (≥60 years) living in LTC facilities, including nursing homes, long-term care homes and comparable residential/service housing settings with 24 h care and on-site meal provision.	People in hospitals, clinics, community dwellings, assisted-living arrangements without on-site catering, or other non-comparable settings.
Concept	Studies evaluating the nutritional adequacy or nutritional quality of food provision in LTC (planned menus, food served and/or residents’ actual intake).	Studies without quantitative nutritional analysis, or focused solely on satisfaction, perception, organisational processes or mealtime experience without energy/nutrient data
Study design	Observational quantitative studies (cross-sectional or longitudinal) reporting primary data with dietary/nutritional analysis.	Non-systematic reviews, commentaries, editorials, duplicate publications, and intervention trials without baseline or outcome data on menu or intake adequacy.
Methodology	Studies using a clearly described dietary assessment method (e.g., weighed food records, plate-waste assessment, menu analysis, food diaries, 24 h recalls or FFQs) allowing quantitative estimation of energy and/or nutrient intake.	Studies with insufficient data or without a clear description of the dietary assessment method.
Comparator	Comparison with official dietary guidelines or nutrient reference values (e.g., DRIs, EFSA DRVs, NNR, ESPEN, or national dietary guidelines).	Studies with no reference to official nutritional recommendations or nutrient reference frameworks.
Source	Studies published in indexed peer-reviewed journals.	Non-peer-reviewed literature, reports, theses or other grey literature.
Language	English or Spanish.	Other languages.
Year of publication	From 2004 to 2024	Studies prior to 2004 or after 2024.

Note: LTC, long-term care; DRIs, Dietary Reference Intakes; EFSA DRVs, European Food Safety Authority Dietary Reference Values; NNR, Nordic Nutrition Recommendations; ESPEN, European Society for Clinical Nutrition and Metabolism. In this review, “nutritional analysis” was defined as any quantitative estimation of the energy and/or nutrient content of foods, menus or dietary intake, as detailed in Section 2.3. Eligibility window: publication years 2004–2024; languages: English and Spanish; source type: peer-reviewed journal articles only. Full electronic search strategies are provided in Supplementary Table S2, and the PRISMA 2020 checklist in Supplementary Table S1.

**Table 2:** Characteristics and comparative synthesis of included studies.

Study (First Author; Year; Country; Setting; *n* [Ref])	Dietary Assessment Method and Level (Days)	Nutritional Adequacy (Summary Codes)	Comparator Framework(s)	JBI/8
Grieger et al., 2007, Australia; NH (*n* = 169) [12]	Visual plate-waste assessment (served and consumed; 3 d)	Mic ↓: Ca, Zn, Fol, vit E, vit B6	ANZ NRVs; DRIs (IOM)	6
Aghdassi et al., 2007, Canada; NH (*n* = 407) [13]	Weighed food record (consumed; 3 d)	E ↓; PRO ↓; Mic ↓: Ca, Mg, Zn, Fol	DRIs (IOM)	6
Lengyel et al., 2008, Canada; LTC centres (*n* = 48; 5 centres) [14]	Weighed food record (consumed; 3 d)	E ↓; PRO ↓; Mic ↓: vit D, Ca; fibre ↓	DRIs (IOM)	6
Woods et al., 2009, Australia; NH (14 homes; *n* = 103) [15]	Weighed food record (consumed; 3 d)	E ↓; PRO ↓; fibre ↓; SFA ↑; Mic ↓: Ca, Mg, Fol, Zn	DRIs (IOM)	7
Massourlard et al., 2010, France; NH (4 homes; *n* = 87) [16]	Weighed food record (consumed; 1 d)	E ↓	National guidelines (France)	6
Beck et al., 2010, Denmark; institutional kitchens (*n* = 4 kitchens) [1]	Weighed food record (planned menus; days NR)	E ↓; PRO ↓	National guidelines (Denmark)	6
Rumbak et al., 2010, Croatia; NH (*n* = 339) [17]	24 h dietary recall (consumed; 1 d)	Mic ↓: Ca	DRIs (IOM)	6
Wright-Thompson et al., 2011, Canada; NH (*n* = 250) [18]	Documentary menu assessment (planned menus; cycle NR)	Mic ↓: vit D, Ca	CFG (Canada); DRIs (IOM)	5
Vikstedt et al., 2011, Finland; service housing (*n* = 375) [19]	Food diary (consumed; days NR)	E ↓ (46% < 1570 kcal/d); PRO ↓ (47% < 60 g/d); Mic ↓: vit D, vit E, Fol, fibre	NNR	6
López-Contreras et al., 2012, Spain; NH (7 homes; *n* = 213) [20]	Weighed food record (consumed; 4 d)	E ↓ (42% < ref)	DRIs (IOM)	6
Milà et al., 2012, Spain; NH (4 homes; *n* = 62) [21]	Weighed food record (consumed; 21 d)	E ↓ (44% < 30 kcal/kg/d); PRO ↓ (10–13% < 0.8 g/kg/d)	National guidelines (Spain)	6
Viveky et al., 2013, Canada; LTC menus (7 d cycle) [22]	Documentary menu assessment (planned menus; 7 d)	Mic ↓: vit D, vit E; Na ↑	CFG (Canada); DRIs (IOM)	6
Lam et al., 2015, Canada; NH (5 homes; menus) [23]	Documentary menu assessment (planned menus; 7 d)	Mic ↓: vit D, vit E, Fol, Mg, K	CFG (Canada); DRIs (IOM)	6
Ongan et al., 2015, Türkiye; NH (25 homes; *n* = 554) [24]	24 h dietary recall (consumed; 1 d)	E ↓; Mic ↓: Ca, Mg, vit A, B1, B2, B6, C, Fol; Fe ↓; Zn ↓	DRIs (IOM)	6
Engelheart et al., 2015, Sweden; NH (*n* = 127) [25]	Weighed food record (consumed; 5 d)	E ↓ (16% < 20 kcal/kg/d); Mic ↓: vit D, Fe	NNR	6
Rodríguez-Rejón et al., 2017, Spain; NH (3 homes; 518 dishes) [26]	Weighed food record (served dishes; 14 d)	Mic ↓: fibre, K, Mg, I, vit D, vit E, Fol	DRIs (IOM)	6
Keller et al., 2017, Canada; NH (32 homes; *n* = 639) [27]	Weighed food record (consumed; 3 d)	E ↓; PRO ↓; Mic ↓: fibre, Ca, Mg, vit D, vit E, Fol	DRIs (IOM)	**8**
Vucea et al., 2017, Canada; NH/LTC menus (7 d cycle) [28]	Documentary menu assessment (planned menus; 7 d)	Mic ↓: vit D, vit E, Ca, Fol	DRIs (IOM)	**8**
Buckinx et al., 2017, Belgium; NH (2 homes; *n* = 74) [29]	Weighed food record (consumed; 5 d)	E ↓; PRO ↓	National guidelines (Belgium)	**8**
Assis et al., 2018, Brazil; NH (29 homes; *n* = 216) [30]	Weighed food record (consumed; 2 d)	E ↓ (30–35 kcal/kg/d); Mic ↓: vit E, Fol, Ca	DRIs (IOM)	6
Nanayakkara et al., 2019, New Zealand; NH (*n* = 54) [31]	Weighed food record (consumed; 3 d)	E ↓ (73% of planned energy); PRO ↓ (69% of planned protein); Mic ↓: vit C, vit B12, Fol	ANZ NRVs; DRIs (IOM)	**8**
Rodríguez-Rejón et al., 2019, Spain; NH (3 homes; *n* = 249) [7]	Weighed food record (consumed; 7 d)	PRO ↓ (56% < 1.0 g/kg/d; 100% < 25 g/meal)	DRIs (IOM); ESPEN/PROT-AGE	**8**
Buckinx et al., 2019, Belgium; NH (*n* = 25) [32]	Weighed food record (consumed; 3 d)	E ↓ (52% deficit)	EFSA DRVs; WHO	6
Carrier et al., 2019, Canada; NH (32 homes; *n* = 619) [33]	Weighed food record (consumed; 3 d)	E ↓; PRO ↓; Mic ↓: vit D, vit E, Fol, Ca, Mg	DRIs (IOM)	**8**
Bogacka et al., 2019, Poland; NH/LTC institutions (79 facilities) [34]	Documentary menu assessment (planned menus; 10 d) + 24 h dietary recall (consumed; 1 d)	Mic ↓: Ca, Mg, vit D; Na ↑; P ↑	EFSA/DRVs; WHO	5
Sossen et al., 2021, Australia; NH (*n* = 420) [35]	Weighed food record (consumed; days NR)	E ↓; PRO ↓ (62–64% of served energy and protein)	ESPEN; ADG; ANZ NRVs	**8**
Grajek et al., 2022, Poland; NH (58 homes; 4640 menus) [36]	Weighed food record (planned menus; days NR)	E ↓; PRO ↓; Mic ↓: vit D, vit A, vit E, Ca, K; refined CHO ↑	DRIs (IOM); WHO	6
Jyväkorpi et al., 2022, Finland; LTC facilities (service housing/NH; 2 cohorts; *n* = 860) [10]	Food diary (consumed; 1–2 d)	E ↑; fat quality ↑ (MUFA/PUFA)	NNR	**8**
Pfisterer et al., 2023, Canada; NH/LTC facilities (*n* = 634) [37]	Weighed food record (consumed; 3 d)	E ↓; fibre ↓ (12.7 g/d)	CFG (Canada)	6
Farapti et al., 2023, Indonesia; NH (*n* = 65) [38]	Visual plate-waste assessment (served and consumed; days NR)	E ↓ (1300 kcal/d); PRO ↓ (50 g/d); fibre ↓; K ↓; Na ↑	National guidelines (Indonesia RDA)	6
Lavriša et al., 2024, Slovenia; NH (20 homes; *n* = 317) [39]	24 h dietary recall + FFQ (consumed; 1 d)	PRO ↓ (subset); fibre ↓; SFA ↑ (30–34 g/d)	DGE; EFSA DRVs	6
Farapti et al., 2023, Indonesia; NH (menus; 5 d cycle; *n* = 61 residents) [40]	Direct menu observation (planned menus; 5 d)	E ↓ (69% RDA); PRO ↓ (66% RDA); Ca ↓; fibre ↓; Na ↑	DRIs (IOM)	6
Li et al., 2024, Australia; NH (60 homes; *n* = 572) [41]	Visual plate-waste assessment (served and consumed; days NR)	PRO ↓ (0.78–0.85 g/kg/d; 20% meals ≥ 25 g protein)	DRIs (IOM)	**8**
Piedrafita et al., 2024, Spain/Portugal; NH (*n* = 186) [42]	Weighed food record (consumed; 7 d)	E ↓ (>50% residents); PRO ↓ (0.97 g/kg/d, Portugal); CHO 56–61%E	EFSA DRVs; WHO	6

Notes. Abbreviations: NH, nursing home; LTC, long-term care; E, energy; PRO, protein; Mic, micronutrients; fibre, dietary fibre; SFA, saturated fatty acids; CHO, carbohydrates; d, day(s); NR, not reported. DRIs (IOM), Dietary Reference Intakes (Institute of Medicine); EFSA DRVs, European Food Safety Authority Dietary Reference Values; NNR, Nordic Nutrition Recommendations; ANZ NRVs, Australia/New Zealand Nutrient Reference Values; CFG (Canada), Canada’s Food Guide; ADG, Australian Dietary Guidelines; DGE, Deutsche Gesellschaft für Ernährung; WHO, World Health Organization; ESPEN/PROT-AGE, European Society for Clinical Nutrition and Metabolism/PROT-AGE Study Group; National guidelines (Country), national nutrient- or food-based recommendations issued by the corresponding health authorities (for Indonesia, National guidelines (Indonesia RDA)). Arrows indicate deviation from the comparator framework: “↓” = below; “↑” = above. Percentages, cut-offs and ranges are reported as in the original articles and may reflect different reference units, age groups or sex strata across studies.

## Data Availability

The raw data supporting the conclusions of this article will be made available by the authors without undue reservation due to privacy and ethical reasons, as the data contains sensitive information from study participants.

## Supplementary Materials

The following supporting information can be downloaded at: https://www.mdpi.com/article/10.3390/nu18010054/s1. Table S1: PRISMA 2020 Checklist. Table S2: Search Strategy.
