# Supplementary material for: Nutritional Adequacy and Dietary Assessment Approaches in Institutionalised Older Adults Living in Long-Term Care Settings: A Systematic Review (2004–2024)
Source: Nutrients. 2025 Dec 23;18(1):54. doi: 10.3390/nu18010054 (PMC12788021; doi:10.3390/nu18010054)
Supplement: Supplementary file 1 [file nutrients-18-00054-s001.zip › Supplementary_Table_S2_Search_Strategy_revised2.pdf]

Supplementary Table S2. Search Strategy

PubMed	Scopus
(("Aged"[Mesh] OR aged [Title/Abstract] OR elderly [Title/Abstract] OR "older adult*"[Title/Abstract] OR geriatric [Title/Abstract])) AND (("Nursing Homes"[Mesh] OR "Skilled Nursing Facilities"[Mesh] OR "Long-Term Care"[Mesh] OR "care home*"[Title/Abstract] OR "nursing home*"[Title/Abstract] OR "residential facility*"[Title/Abstract] OR "residential care"[Title/Abstract] OR "skilled nursing facility*"[Title/Abstract])) AND (("Food Services"[Mesh] OR "Food Service, Hospital"[Mesh] OR "menu planning"[Title/Abstract] OR "meal plan*"[Title/Abstract] OR "menu assess*"[Title/Abstract] OR "nutritional adequacy"[Title/Abstract] OR "menu quality"[Title/Abstract] OR "dietary assessment"[Title/Abstract] OR "nutrient intake"[Title/Abstract] OR "reference values"[Title/Abstract] OR "diet quality"[Title/Abstract] OR "dietary guidelines"[Title/Abstract] OR "plate waste"[Title/Abstract] OR "food consumption"[Title/Abstract] OR "weighed food record"[Title/Abstract] OR "24-hour recall"[Title/Abstract] OR "food diary"[Title/Abstract] OR "FFQ"[Title/Abstract] OR "indirect calorimetry"[Title/Abstract]))	(TITLE-ABS-KEY (aged OR elderly OR "older adult*" OR geriatric)) AND (TITLE-ABS-KEY ("nursing home*" OR "care home*" OR "residential facility*" OR "long-term care" OR "residential care" OR "skilled nursing facility*")) AND (TITLE-ABS-KEY ("food service*" OR "menu planning" OR "meal plan*" OR "menu assess*" OR "nutritional adequacy" OR "menu quality" OR "dietary assessment" OR "nutrient intake" OR "reference values" OR "diet quality" OR "dietary guidelines" OR "plate waste" OR "food consumption" OR "weighed food record" OR "24-hour recall" OR "food diary" OR "FFQ" OR "indirect calorimetry"))
